# Spanlastic Nano-Vesicles: A Novel Approach to Improve the Dissolution, Bioavailability, and Pharmacokinetic Behavior of Famotidine

**DOI:** 10.3390/ph17121614

**Published:** 2024-11-29

**Authors:** Hend I. Almohamady, Yasmin Mortagi, Shadeed Gad, Sawsan Zaitone, Reem Alshaman, Abdullah Alattar, Fawaz E. Alanazi, Pierre A. Hanna

**Affiliations:** 1 Department of Pharmaceutics, Faculty of Pharmacy, Sinai University, Arish 45511, Egypt; hend.ibrahem@su.edu.eg (H.I.A.); yasmin.mohamed@su.edu.eg (Y.M.); 2 Department of Pharmaceutics and Industrial Pharmacy, Faculty of Pharmacy, Suez Canal University, Ismailia 41522, Egypt; shaded_abdelrahman@pharm.suez.edu.eg; 3 Department of Pharmacology and Toxicology, Faculty of Pharmacy, University of Tabuk, Tabuk 71491, Saudi Arabia; ralshaman@ut.edu.sa (R.A.); aalattar@ut.edu.sa (A.A.); falanazi@ut.edu.sa (F.E.A.); 4 Department of Pharmaceutical Technology, Faculty of Pharmacy, Misr International University, Cairo 12585, Egypt

**Keywords:** spanlastics, biphasic release, membrane permeability, famotidine, bioavailability

## Abstract

**Background/Objectives**: Drugs exhibiting poor aqueous solubility present a challenge to efficient delivery to the site of action. Spanlastics (a nano, surfactant-based drug delivery system) have emerged as a powerful tool to improve solubility, bioavailability, and delivery to the site of action. This study aimed to better understand factors affecting the physicochemical properties of spanlastics, quantify their effects, and use them to enhance the bioavailability of famotidine (FMT), a model histamine H2 receptor antagonist (BCS class IV). **Methods**: FMT was incorporated into nano-spanlastics drug delivery system. The ethanol injection method, Box–Behnken design, and mathematical modeling were utilized to fabricate famotidine-loaded nano-spanlastics and optimize the formula. Spanlastics were characterized for their particle size, polydispersity index, zeta potential, entrapment efficiency, drug loading, compatibility of the excipients (using DSC), in vitro drug release, and in vivo pharmacokinetics. **Results**: Span 60 (the non-ionic surfactant) and tween 60 (the edge activator) gave rise to spanlastics with the best characteristics. The optimal spanlastic formulation exhibited small particle size (<200 nm), appropriate polydispersity index (<0.4), and zeta potential (>−30 mV). The entrapment efficiency and drug loading of the optimum formula assured its suitability for hydrophobic drug entrapment as well as practicability for use. DSC assured the compatibility of all formulation components. The drug release manifested a biphasic release pattern, resulting in a fast onset and sustained effect. Spanlastics also showed enhanced C_max_, AUC_0–24_, and bioavailability. **Conclusions**: Spanlastics manifested improved FMT dissolution, drug release characteristics, membrane permeation, and pharmacokinetic behavior.

## 1. Introduction

The poor aqueous solubility of nearly 90% of recently developed drugs (new chemical entities, NCE) and about 40% of well-established drug molecules poses a significant obstacle to their use as active agents. For any active ingredient to exert a pharmacological effect, it needs to be absorbed and reach the general circulation in an appropriate concentration. This could only take place after the drug is soluble in the aqueous environment of the GIT [1]. Therefore, aqueous solubility becomes a significant barrier to the systemic use of large drug molecules. The biopharmaceutics classification system (BCS) categorizes drugs with poor aqueous solubility under classes II and IV [2].

Many strategies have been adopted extensively to enhance the aqueous solubility of the drugs exhibiting poor aqueous solubility (BCS II and IV drugs). These strategies comprise physical and chemical drug changes and include the modification of the chemical structure of the drug molecule to impart hydrophilicity to it [3], the use of eutectic solvent systems [4], the engineering of the drug crystal [5], the engineering of a drug carrier system [6], reducing the particle size of the drug particles [7], solubilization [8], and the use of a drug nanocarrier system that imparts new physicochemical characteristics to the drug molecule [9].

The oral delivery of BCS class IV drugs faces more challenges than the delivery of BCS class II drugs since solubility is not the only hurdle. The permeability of BCS class IV drugs is also as poor as their solubility [10]. This implies special importance to the nanoparticulate drug delivery systems because they not only increase solubility (an approach that seems enough for the delivery of BCS class II drugs) but also increase the permeability of the entrapped drugs [11,12].

Among the recently utilized nanocarrier drug delivery systems arise vesicular nanocarriers as advantageous carriers for lipophilic, hydrophilic, and amphiphilic drugs [13]. These include liposomes, invasomes, niosomes, and spanlastics [14]. Spanlastic nano-vesicles (SNVs) emerge as a surfactant-based nanocarrier drug delivery system that not only enhances solubility but also improves the penetration of target drugs to specific sites or organs and ameliorates the pharmacokinetic behavior of the drug molecules [15,16,17,18]. Although Kakkar and Kaur first established them as ocular drug delivery systems in 2011, they have now explored applications for the delivery of drugs to a variety of other body regions. The spanlastics bilayers’ elasticity and deformability are increased by the presence of an edge activator (EA), which enhances drug absorption through biological membranes [19].

Considering that polar and non-polar components make up nano-vesicular systems, they have the ability to entrap lipophilic and hydrophilic medicines [20]. The stability and permeability of medications that have been encapsulated can both be enhanced by nan-vesicular systems. Moreover, they could deliver medicines in a controlled or steady manner to their target areas, increasing their bioavailability [21].

Famotidine (FMT) is a popular prokinetic medication and competitive H2 receptor antagonist having the chemical formula C8H15N7O2S3 and is known by the IUPAC name 3-[[2-(diaminomethylideneamino)-1,3-thiazol-4-yl]methylsulfanyl]-N-sulfamoylpropanimidamide [22]. The suppression of stomach acid secretion is its primary pharmacodynamic action. When taken orally (20 mg or 40 mg), it accelerates the healing of duodenal ulcers and reduces stomach acid production by up to 90%. Heartburn, ulcers, and esophageal inflammation are all diseases it is used to treat. Zollinger–Ellison syndrome is treated with large doses of the drug [23]. FMT is identified as a limited solubility (1 mg/mL) and poor permeability drug, making it a good model drug for BCS class IV drugs [24]. Due to FMT’s short biological half-life (2.5–3.5 h) and low oral bioavailability (40–45%), a sustained release formulation has a greater chance of being beneficial [25]. It was reported that the aqueous solubility and oral bioavailability of FMT have been improved through the use of niosomes, solid–lipid nanoparticles, cyclodextrin complexation, solid dispersion, cocrystals, and S-SNEDDS, among other methods [25]. However, the possibility of encapsulating and delivering FMT in spanlastic nano-vesicles has not yet been investigated. Famotidine was also reported to be degraded in the gastric juices, rationalizing the use of buccal and sublingual routes to enhance its delivery [25].

Sublingual and buccal routes of drug administration have been utilized for many years to improve the bioavailability of drugs suffering first-pass metabolism and/or gastric juice degradation [26]. Compared to the conventional oral route of administration, sublingual routes exhibit higher bioavailability as well as a faster onset of action [26,27]. This is made possible by the region’s enriched blood supply as well as the direct entry into general blood circulation, avoiding stomach and mesenteric veins and hence acid-catalyzed degradation and first-pass metabolism [26,27]. A great challenge facing the administration of the drugs through the sublingual route is that this region is affected by saliva. The rate of drug dissolution is affected by saliva volume and composition [28]. Drug administration via the sublingual route is also affected by the tongue movement, making it a challenge towards the insertion of solid controlled drug delivery systems in the sublingual area. Up to the date of reporting this study, no sublingual spanlastic formulations have been reported to be developed; only spanlastics for the buccal route of administration were reported [29].

The aim of this study was to design and develop a superior drug delivery system that overcomes hurdles facing the delivery of BCS class IV drugs (poor solubility and permeability), protects the cargo from gastric degradation, and at the same time exhibits a desired release pattern of the drug to give rapid onset of action with a sustained effect. This could be achieved through gaining a better knowledge of the factors affecting the physicochemical properties of the advantageous and novel nano-spanlastics. The optimization of the drug delivery system, then, could be achieved in order to determine the appropriate delivery and pharmacokinetic patterns of the drug. FMT was used as a model drug, and an evaluation of the nano-spanlastics system was carried out.

## 2. Results

### 2.1. Preparation of SNVs

The design of the experiment revealed 15 formulations to be prepared. The dependent variables selected for determination were particle size (PS), the polydispersity index (PDI), zeta potential (ζ), entrapment efficiency (EE), and percentage drug loading (DL). The obtained values of these variables are listed in Table 1.

### 2.2. Effect of Independent Variables on PS

The mean PS of the SNV dispersions ranged from 89.04 ± 0.57 nm (F5) to 282.20 ± 6.22 nm (F12). It was observed that increasing the concentration of span 60 (S 60) had a positive impact on the mean PS of SNVs. This was clearly illustrated by Equation (1). On the contrary, the mean vesicle size and the edge activator (EA) concentration were shown to have inverse correlations, i.e., vesicles formed at the highest EA concentration manifested the smallest PS. This came along with the findings reported in other studies [30,31]. The amount of FMT incorporated inside the SNVs was shown to urge the particle size to increase. This was clearly pronounced by the positive value of the slope of the term “C” in Equation (1).

(1) $$PS=163.86+22.02\;A-5.42\;B+74.85\;C+3.63\;AB+10.22\;AC-1.25\;BC-0.5579\;A^{2}+2.36\;B^{2}+12.09\;C^{2}$$

where A is the span concentration (mg/mL), B is the concentration of tween 60 (mg/mL), and C is the amount of FMT originally incorporated in the SNVs.

**Figure 1:**
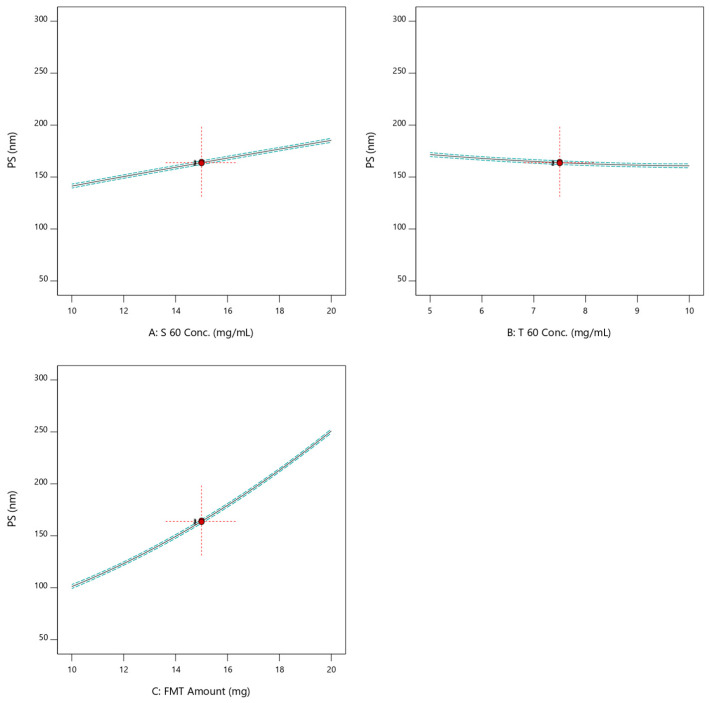
Linear plots of the effects of individual formulation variables on the PS of SNV.

The separate effects of the formulation variables on the PS of the nano-vesicles were illustrated by Figure 1. It was demonstrated that the positive slopes of S 60 concentration and FMT incorporated amount indicated that they had a positive impact on the PS. The higher slope of the curve of FMT amount indicated that the latter exhibited a greater impact on the PS than the former. This could also be observed from the higher value of the factor multiplied by the FMT amount as compared to the lesser one multiplied by the S 60 concentration in Equation (1). On the other hand, the negative slope of the curve describing the individual effect of tween 60 (T 60) on the PS reveals that they are slightly inversely correlated.

**Figure 2:**
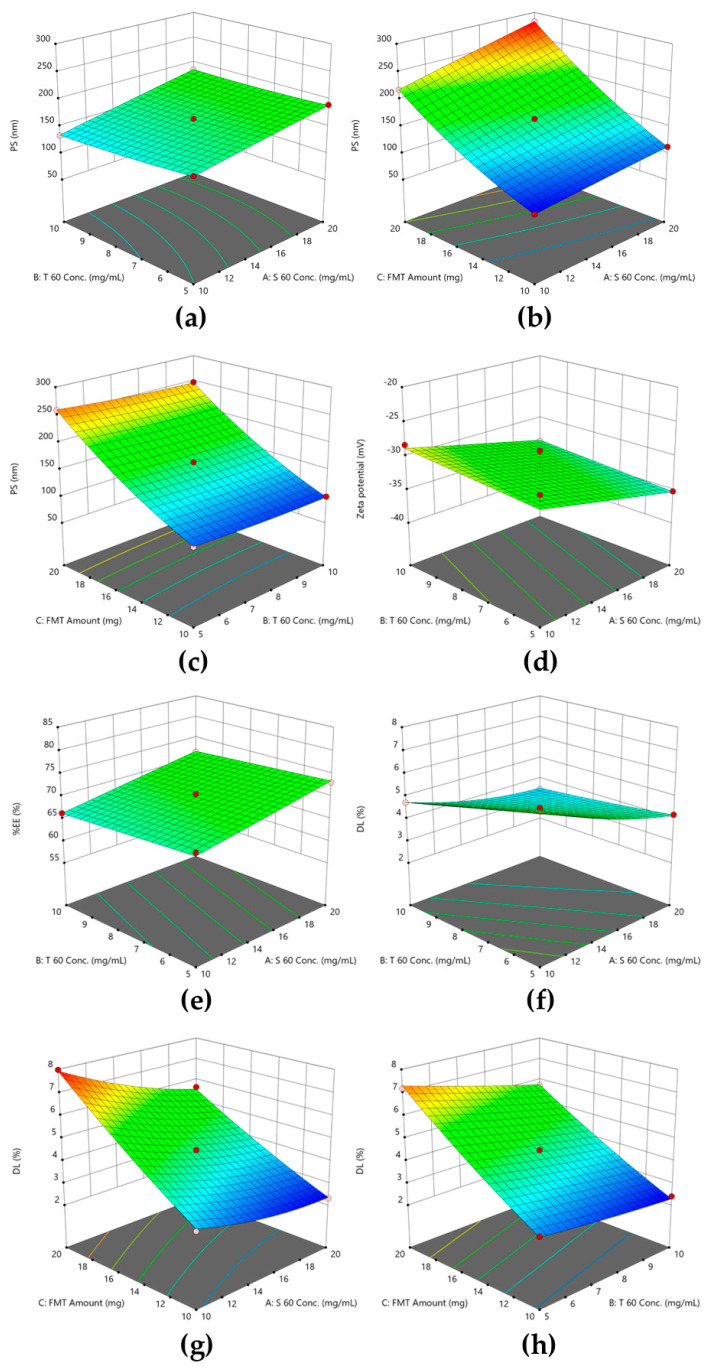
Three-dimensional plots of the combined effects of independent variables (**a**) S 60 and T 60 concentrations on the PS; (**b**) S 60 concentration and FMT amount on the PS; (**c**) T 60 concentration and FMT amount on the PS; (**d**) S 60 and T 60 concentrations on ζ; (**e**) S 60 and T 60 concentrations on EE; (**f**) S 60 and T 60 concentrations on DL; (**g**) S 60 concentration and FMT amount on DL; (**h**) T 60 concentration and FMT amount on DL.

The combined effect of each of the two formulation variables was illustrated by the contour plots of these effects in Figure 2. The effect of independent variables on the PS was shown to follow the quadratic mathematical model. This indicated that the combination of these variables would have an optimum composition to prepare SNVs with the minimum possible PS.

### 2.3. Effect of Formulation Variables on PDI

As shown in Table 1, the polydispersity index of all the formulations tested in the current study ranged from 0.346 ± 0.02 to 0.493 ± 0.03. In addition, no single mathematical model fitted the values of the PDI in correlation with the three independent variables used. The *p*-values of the lack of fit of this design to all mathematical models ranged from 0.0620 to 0.0738. This indicated that the correlation between formulation variables and PDI values was not statistically significant, i.e., the PDI was not affected by the change of formulation variables in a distinct manner.

### 2.4. Effect of Formulation Variables on ζ

It was clearly evidenced that the EA had a minor effect on the magnitude and the charge of ζ. On the other hand, drug amount showed the highest effect on the ζ of the SNVs. The concentration of S 60 affected the nano-vesicles’ zeta potential; however, this effect was minor compared to the effect of the amount of FMT incorporated in the vesicle. It was shown by Table 1 that increasing the S 60 concentration from 10 to 20 mg/mL while maintaining the other variables at the same levels (F3 and F12, respectively) caused the magnitude of zeta potential to rise from −35.93 ± 3.33 to −39.77 ± 0.77 mV (*p* < 0.05). In the same way, ζ increased in value from −28.07 ± 0.80 to −39.77 ± 0.77 mV upon increasing the FMT amount from 10 to 20 mg (F8 and F12, respectively). This was also illustrated by Figure 2d, where the slope of the curve was steeper in relation to the panel of the FMT amount compared to the panel of the S 60 concentration. The effect of S 60 concentration and FMT amount on ζ followed linear mathematical modeling, as shown by Figure 2d. The equation best describing this effect is listed below (Equation (2)).

(2) ζ=−32.04−2.40 A+0.8150 B−5.62 C

### 2.5. Effect of Different Formulation Variables on EE and DL

**Figure 3:**
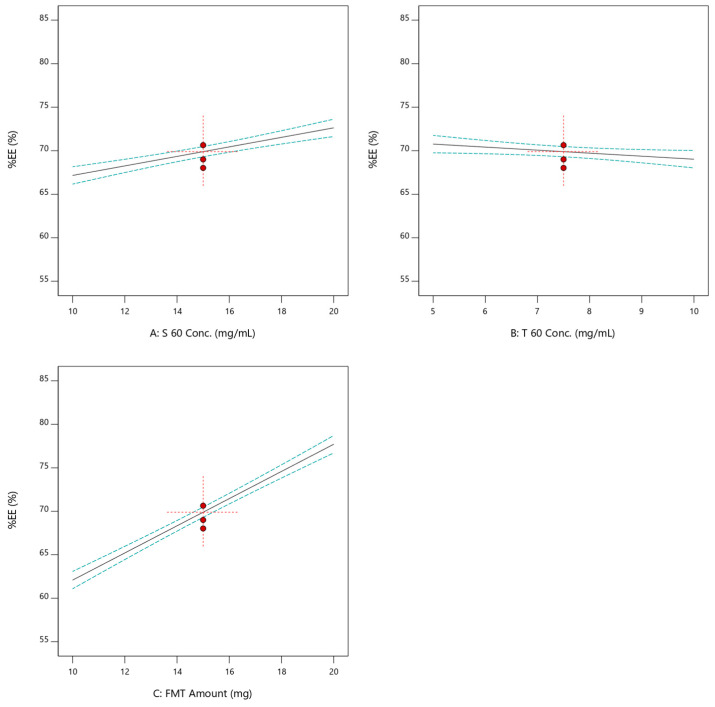
Linear plots of the effects of individual independent variables on the EE of SNV.

The effect of formulation variables on EE was shown to follow a linear model (Figure 2e). As illustrated by Figure 3, EE was detected to be positively correlated with the S 60 concentration as well as the FMT amount. On the contrary, as the concentration of T 60 increased, the EE decreased. The formulation F12, which contained a high concentration of span 60 (20 mg/mL), displayed an EE of 82.32% (Table 1), while F5, which contained the lowest amount of span 60 (10 mg/mL), showed a decline in EE (59.37%). As was discussed for ζ, the effect of the FMT amount was higher than that of the S 60 concentration. This was demonstrated by the steep slope of the linear plot describing the effect of the FMT amount on EE (Figure 3). Mathematical equation deduced from the analysis of the effect of formulation parameters on EE is listed below (Equation (3)). How well a statistical model fits a set of observations is known as its goodness of fit. Measures of goodness of fit are frequently used to summarize the difference between actual values and the values predicted by the in-question model. The linear model was chosen as the following model for evaluating the obtained EE values according to an ANOVA statistical analysis. The Design-Expert program decided that the measurement of the ratio of signal to noise had adequate precision; a ratio >4 is ideal. The study model’s accuracy was 39.52, and there was a reasonable level of agreement between the adjusted R^2^ (0.974) and expected R^2^ (0.964). This might contribute to the reliability of the model that was used to explore the design space [31].

(3) $$EE\;\left(\%\right)=69.98+2.73\;A-0.8688\;B+7.82\;C\;$$

**Figure 4:**
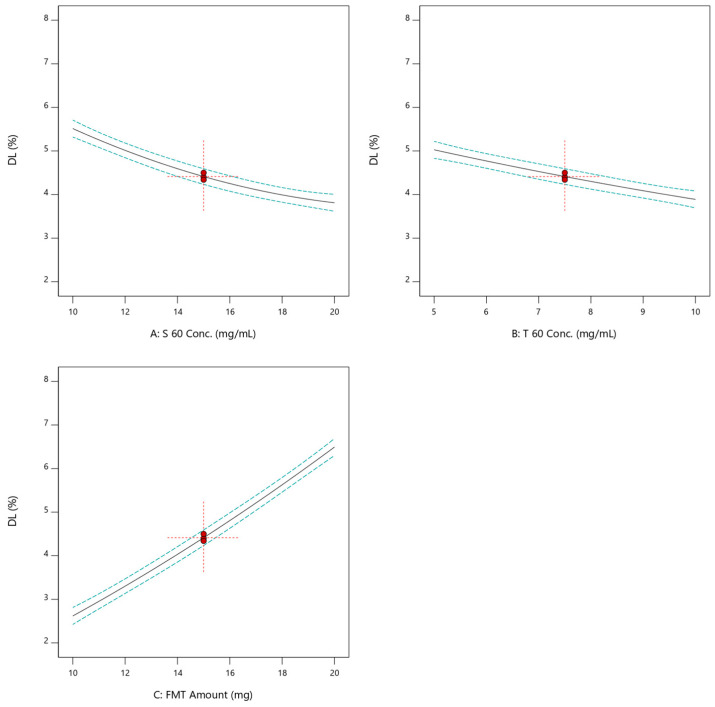
Linear plots of the effects of individual independent variables on the DL of SNV.

The drug loading of all formulations prepared in this study ranged between 2.31 and 7.98. For a drug that is used in a small dose like FMT, this range is accepted [32]. Figure 4 illustrated that S 60 and T 60 concentrations, on the contrary to the FMT amount, had a negative impact on the DL of FMT. The correlation between independent variables and DL was shown to follow a quadratic mathematical model (Figure 2f–h). The mathematical equations that best described the value of DL as a function of S 60 and T 60 concentrations and FMT amount are listed below:

(4) $$DL\;\left(\%\right)=4.10958-0.40825\;A-0.3855\;B+0.538\;C+0.019\;AB-0.0136\;AC-0.0156\;BC+0.009983\;A^{2}+0.007133\;B^{2}+0.005683\;C^{2}$$

### 2.6. Optimization of SNV

**Figure 5:**
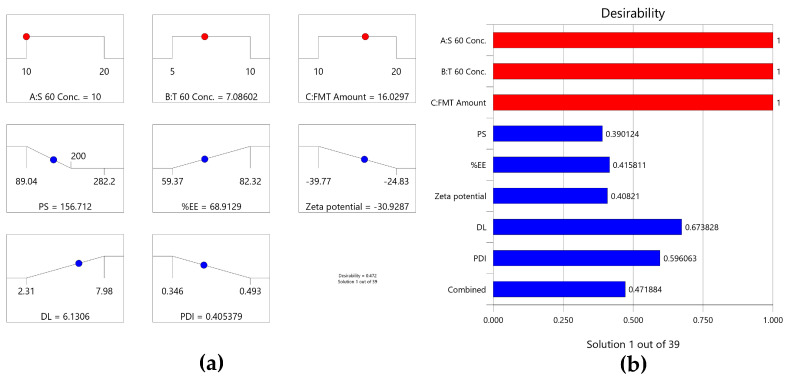
(**a**) Ramp graphs for the optimized variable levels and predicted responses of the optimized SNV (FOSNV); (**b**) bar graph for the desirability of the optimization process.

Predicting the levels of variables that will result in a formulation with the desired physicochemical characteristics is the primary goal of pharmaceutical optimization. To create the optimum FMT-loaded SNV formulation (FOSNV) with the lowest PS and PDI and the highest zeta potential, EE, and DL, this work used numerical optimization. Based on the analysis of the effects of all independent variables on the physicochemical properties of the SNVs and setting the desirable goal of the SNV, 39 formulations were proposed by the software to meet the required optimum physicochemical characteristics. The level of each independent variable and the proposed value of each variable are shown by Figure 5a. The first suggestion (the suggested formula with the highest combined desirability) was selected as the optimum SNV (Figure 5b). The FOSNV was suggested to be prepared using S 60 and T 60 concentrations at levels of 10 mg/mL and 7.09 mg/mL, respectively. The FMT amount incorporated into the FOSNV was suggested to be 16.03 mg. The combined desirability score of the selected FOSNV was 0.472.

### 2.7. Evaluation of Physicochemical Characteristics of FOSNV

Evaluating the physicochemical properties of the FOSNV (Table 2) revealed that all the actual physicochemical characteristics of the FOSNV were close to those predicted by the experimental design. The highest calculated relative error between the actual and the predicted FOSNV characteristics was less than 9% (PS). This indicated that the experimental design was accurate and the mathematical modeling of the correlations between independent and dependent variables was precise.

#### 2.7.1. Particle Size, PDI, and Zeta Potential

The PS and PDI of the FOSNV were measured to be 170.58 ± 4.48 and 0.368 ± 0.04, respectively. This indicated an appropriate particle size with adequate distribution. The zeta potential of the FOSNV was found to be higher than −30 in value (Table 2). This indicated the potential stability of the system with minimum probability for aggregation on storage [33].

#### 2.7.2. Entrapment Efficiency and Drug Loading

As it was listed in Table 2, the FOSNV showed an EE and DL of 68.89 ± 0.48 and 6.07 ± 0.04, respectively. These values indicated that most of the incorporated amount of FMT was trapped in the vesicles. The appropriate value of DL achieves an adequate practical utility of the FOSNV.

#### 2.7.3. Vesicles Deformability

Before extrusion, the particle size of the FOSNV was 170.58 ± 4.48 nm; however, after extrusion, it became 167.14 ± 0.22. This minor change in the PS of the optimized formula gave rise to an excellent DI (8.26 ± 0.18). This indicated good flexibility of the FOSNV.

#### 2.7.4. Differential Scanning Calorimetry

**Figure 6:**
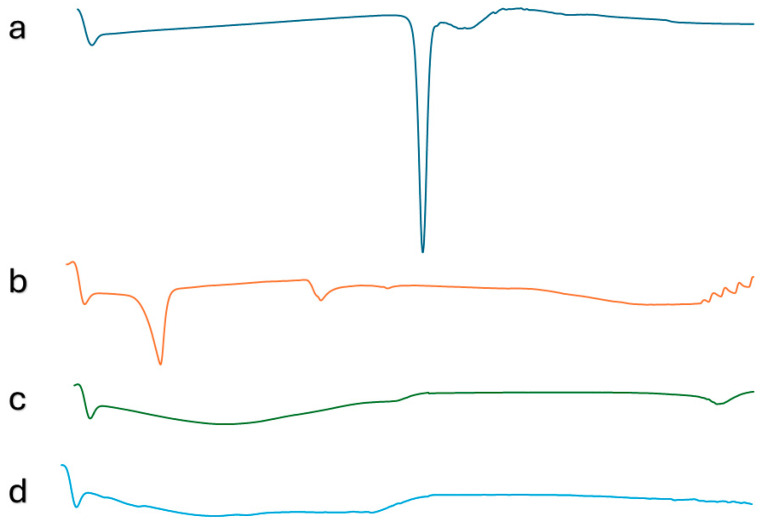
DSC thermograms of (**a**) FMT, (**b**) S60, (**c**) plain optimized SNV, and (**d**) FOSNV.

Figure 6 displayed the DSC thermograms of FMT, S 60, the plain optimized SNV, and the FOSNV. A sharp, well-defined endothermic peak at 166 °C, which corresponds to FMT’s melting temperature, can be identified on the thermogram of FMT (Figure 6a) [25]. In addition, S 60 displayed an endotherm signal at 57.85 °C, which corresponds to the temperature at which the melting process of this substance takes place (Figure 6b). The thermogram of the plain SNV demonstrated the disappearance of the endothermic peak corresponding to the melting of S 60 (Figure 6c). Additionally, the thermogram of the FOSNV (Figure 6d) showed that neither peaks appearing in FMT nor S 60 thermograms appeared in the FOSNV thermogram.

#### 2.7.5. Morphological Characteristics of FOSNV

**Figure 7:**
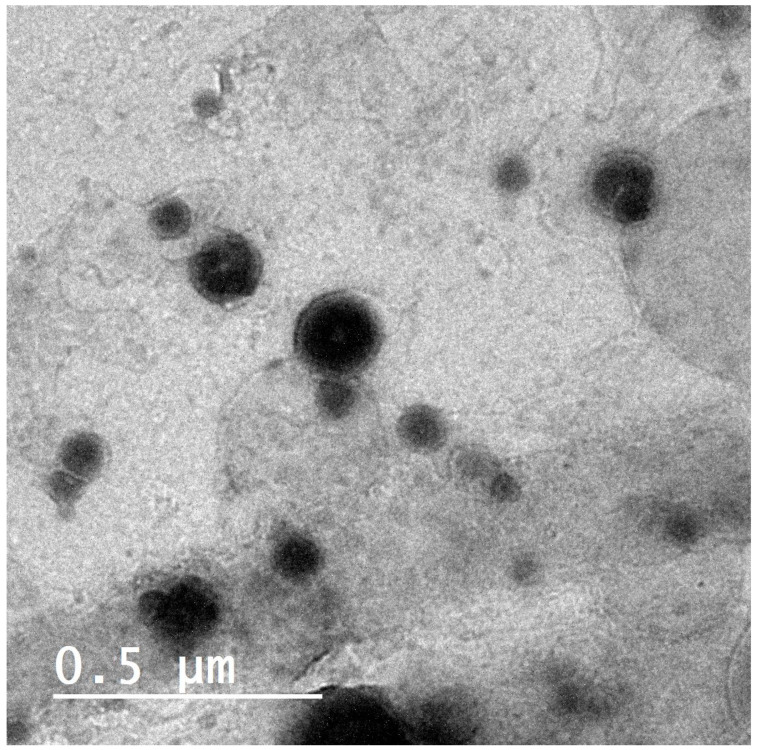
TEM of FOSNV.

The transmission electron micrograph of the FOSNV (Figure 7) showed that the nano-vesicles manifested a smooth surface with a uniform spherical shape, high dispersion, and almost no aggregations. The surfactant-based bilayer of the vesicles was distinctive from the interior of the vesicles. The TEM showed also that the FOSNV was unilamellar vesicles. The size exhibited by this measurement (about 135 nm) was smaller than the size determined by dynamic light scattering measurements (DLS).

#### 2.7.6. In Vitro Drug Release

**Figure 8:**
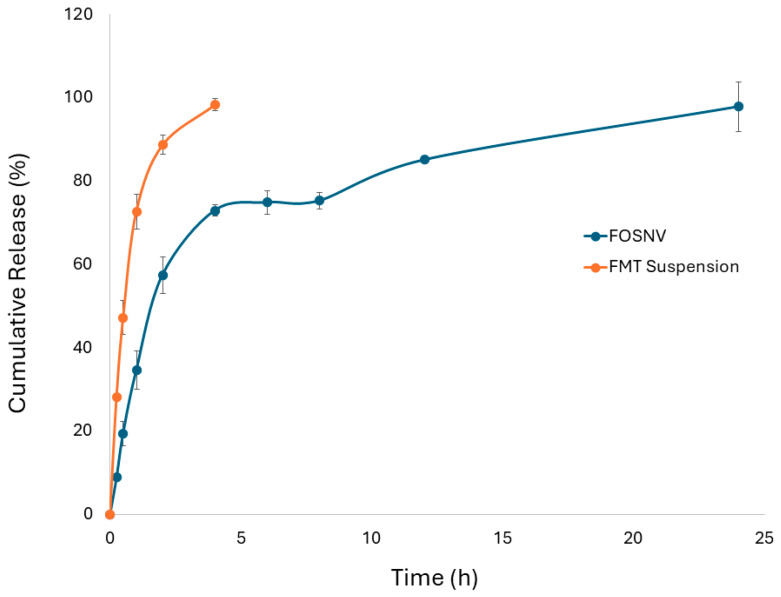
In vitro drug release profiles of FOSNV and FMT suspension.

The in vitro release profile of FMT from the FOSNV compared to the FMT suspension was illustrated by Figure 8. It was demonstrated that the release pattern of FMT from the FOSNV showed biphasic release. A weighable amount of the drug was abruptly released in a short time at the beginning of the study (19.43% ± 2.95 of FMT was released during the first 30 min). This fast release was followed by a controlled release pattern (22.89% of the drug incorporated in the spanlastic nano-vesicles was released for 18 h). About 97.86 ± 6.00% of FMT was released from the FOSNV after 24 h. On the other hand, the release of the FMT suspension was found to be fast. During the first 30 min of the study, 47.24% ± 4.05 of the entrapped amount of the drug was released. In addition, 88.71% ± 2.36 of the drug was released during the first 2 h. After only 4 h, almost 100% (98.39% ± 1.53) of the labeled amount of the drug was released.

### 2.8. In Vivo Pharmacokinetic Study

**Figure 9:**
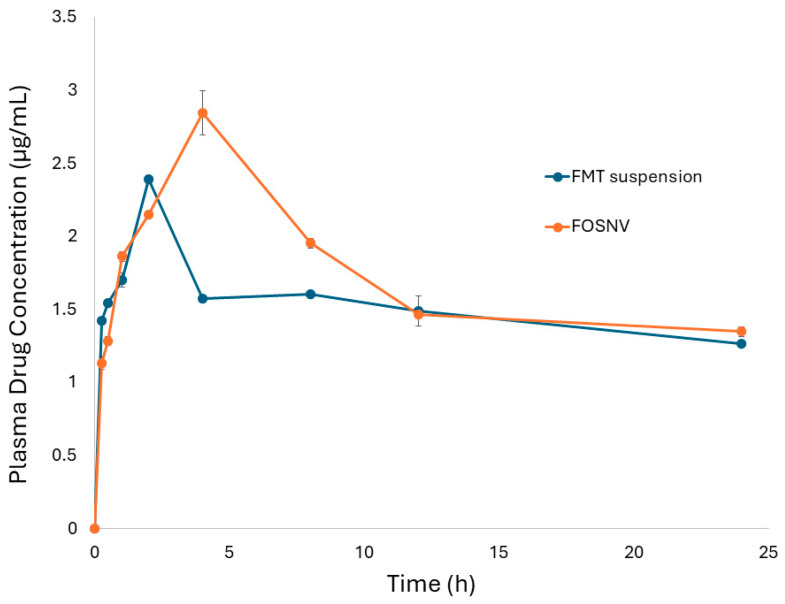
Plasma drug concentration–time profiles of the FOSNV and FMT suspension after the administration of a sublingual liquid single dose.

The plasma drug concentration–time profiles of the FOSNV and FMT pure suspension exhibited different pharmacokinetic patterns (Figure 9). Regarding the FMT pure suspension, the drug reached its maximum concentration (2.394 μg/mL) after 2 h from the drug administration. On the other hand, FMT was detected at its highest concentration in the plasma (2.845 μg/mL) after 4 h of the administration of the FOSNV. The curve of the FOSNV was observed to enclose more area compared to that of the pure FMT suspension.

## 3. Discussion

### 3.1. Preparation of SNV

FMT-loaded SNVs were successfully formulated by the ethanol injection method. The ethanol injection method was reported to be advantageous over other methods used for vesicles preparation, e.g., the thin film hydration method, in that it is easier to perform (one-step-based method), has higher reproducibility, and is capable of creating small nano-vesicles with limited size distribution.

Span 60 was used as the non-ionic surfactant in the current study. The lipophilic saturated alkyl chain of S 60 (HLB = 4.7) promoted the development of stable unilamellar vesicles with good encapsulation efficiency. Due to its ability to condense membranes or to form a phase with interpenetrating hydrocarbon chains, ethanol reduced the size of the vesicles and improved drug partitioning and entrapment within these nano-vesicular spanlastics. It finally altered the net charge of the system towards a negative zeta potential, which resulted in some degree of steric stabilization [34,35,36].

The incorporation of tween 60 as an edge activator is suggested to increase the penetration of the SNV through cells. This was reported to take place as a result of the fact that T 60 temporarily created an increase in the cell membrane pore size, thus even larger vesicles can easily enter the cell cytoplasm through these enlarged pores [36].

### 3.2. Effect of Formulation Variables on Particle Size

The produced dispersions’ decreased tendency for aggregation and smaller diameters than edge activator-free dispersions may be the result of the edge activators’ positive impact on them [35,36,37]. The mean vesicle size and the edge activator concentration were shown to have inverse correlations. The vesicles formed at the highest edge activator concentration are the smallest. [30,31]. This could be attributable to the increased emulsification power that was observed while using higher quantities of the latter. The conclusion that could be displayed is that the lower concentrations of edge activators might not be able to completely cover the vesicle surface [38]. Parallel to that, the presence of more alkyl chains in the lipophilic region of the vesicles, which inhibited the contact among the polar heads of the edge activator molecules, was related to the increase in vesicle size found with higher S 60 concentrations. With T 60-decorated dispersions, considerably (*p* < 0.01) smaller vesicles were obtained with the higher span 60 concentration. This was also confirmed by Ansari et al. that the vesicle size increased if the amount of span 60 increased [39].

### 3.3. Effect of Different Variables on Poly Dispersity Index (PDI)

The PDI is considered to be a measurement of particle population homogeneity. A homogeneous (monodisperse) system is indicated by small PDI values [34]. In order to indicate a narrow size distribution of particles, the PDI value should ideally be less than 0.7, according to Abdellatif et al. [40]. In other words, lower PDI values (<0.5) assured a dispersion formation of consistent distribution for vesicle size. The PDI of the formulations tested in the current study ranged from 0.346 to 0.493, revealing that their size distributions were typically uniform. It was clearly illustrated that PDI values of the SNV were independent of the formulation variables (no mathematical model significantly fitted the data correlating the independent variables to PDI values).

### 3.4. Effect of Different Variables on Zeta Potential (ZP)

Zeta potential has an effect on both the stability of nanoparticles and the efficiency of in vivo drug delivery, making it one of the most crucial parameters for the characterization of nanoparticles [41]. The zeta potential was less extensively affected by the amount of edge activators, according to ANOVA statistics (*p* < 0.05). This was contradictory to the findings reported by Tayel et al., which stated that edge activator concentration had a significant effect (*p* < 0.01) on the zeta potential values [36]. Tayel et al. reported that increasing the EA concentration resulted in increased accumulation of it on the surface of the vesicles, leading to an increase in the shielding effect of the EA against the negative charges of the carboxylic groups of other molecules in the vesicles [36]. However, in this study, pravastatin sodium was utilized as the drug. Pravastatin sodium is an ionic drug with strong charges accumulated on its molecule. Increasing the amount of EAs could result in hindering the ionic moieties of pravastatin sodium from interacting with the external environment, resulting in a great reduction in the surface charges of the vesicles. This could lead to a significant decrease in the ζ. Incorporating FMT (a weakly ionized drug) in the vesicles could lead to different results since the hindering effect would not be as pronounced as that noted with using a strongly ionizable drug. On the other hand, despite having a minor effect on the zeta potential of the nano-vesicles, the EA shows a significant impact on the stability of the nano-vesicles. This could be achieved, not by electrically stabilizing the vesicles but rather by the steric stabilization of the spanlastic nano-vesicles [42].

On the other hand, increasing the amount of S 60 from 100 to 200 mg caused the zeta potential to rise from 35.9 ± 3.3 to 39.8 ± 0.25 mV. The partial negative polar heads of S 60 that faced the aqueous medium are suggested to be responsible for this negative charge, as reported by Mowafy et al. [43]. In the same way, ζ increased from −28.1 ± 0.8 to −39.8 ± 0.25 mV when FMT concentration was increased from 10 to 20 mg. This could be due to a drug charge that has sulfur dioxide and sulfur at the thiazole group of its structure. This interpretation, mentioned before by Tayel et al., who found that PVS has an alkyl chain terminated with a carboxylic acid group and two hydroxyl groups at the b and d positions, may be a contributing factor for these negative charges [36].

### 3.5. Effect of Formulation Variables on EE and DL

Designing FMT spanlastic nano-vesicles with a high EE is challenging. As shown by statistical analysis, the EE of SNVs was significantly impacted by non-ionic surfactant concentration (X1), EA concentration (X2), and FMT concentration (X3) (*p* < 0.05 for all variables). The formulation F12, which contained a high concentration of S 60 (20 mg/mL), displayed an EE of 82.32%, while F3, which contained a lower amount of S 60 (10 mg/mL), showed a decline in EE (75.85%). This result suggested that S 60 is accountable for better drug retention in the vesicle. In addition, having a 4.7 HLB value and a lipophilic nature, S 60 may create a multilamellar matrix in the vesicle at higher concentrations, enhancing EE [36]. However, in this study, the concentrations of S 60 utilized to formulate the FOSNV were not high enough to produce multilamellar SNVs. Tween 60 (the used edge activator) also enhanced the effectiveness of the SNVs to entrap the drug. T 60 enhanced the vesicles’ elastic properties and allowed them to increase the biological membranes’ pore sizes, allowing somewhat larger vesicles to squeeze through enlarged pores and improve drug penetration [36,44]. Additionally, these water-loving surfactants (EA) can destabilize vesicular membranes by altering the packing characteristics of systems to varying degrees, making them more deformable, and enhancing their instability [36,45]. Regarding FMT conc (X3), using a higher conc of FMT (20 mg) led to an EE increase. This could be due to the presence of a higher amount of lipophilic drug in the formulation, which permitted more amount of FMT to be entrapped inside vesicles. The increase in FMT amount, thus, resulted in larger particle size with enhanced entrapment efficiency [36].

It was shown that increasing the amount of the incorporated FMT with reducing amounts of S 60 and/or T 60 resulted in an increased DL of the SNV. This could be attributed to the way DL is calculated. Since DL is calculated by dividing the amount of the drug encapsulated inside the SNV by the whole weight of the nano-vesicle, increasing the FMT amount and/or decreasing the weight of the other components is suggested to result in an increase in the DL value. The DL value represents the ability of the drug delivery system to deliver an appropriate amount of the drug through the administration of an appropriate weight or volume of the formula. This indicates that DL (which differs from EE) is an important key parameter that is related to the ability of the drug delivery system to be used practically [32,46].

### 3.6. Development of FOSNV Formula

The FOSNV was developed as the optimum formulation of an FMT-loaded spanlastic nano-vesicle prepared utilizing components and formulation parameters in this study. The PS was chosen as a crucial parameter for suggesting FOSNV formulation compositions. This could be justified on the basis that the PS affects the penetration capability of nanoparticles and their distribution patterns [47]. FMT is a BCS class IV drug, indicating that the penetration capability of the nanocarrier is a crucial parameter that extensively affects the bioavailability of the drug. EE and DL were also selected as important key parameters that affect the capability of the nanocarrier to deliver FMT in an adequate manner. High entrapment efficiency connotes increased drug concentration and bioavailability, which may lead to a reduction in treatment dose and a lessening of dose-dependent systemic adverse effects [48,49]. However, the importance of EE as a crucial parameter in the optimum SNV formulation design was not as important as the PS. This could be attributed to the finding that all formulations manifested EE values above 60%, which is considered accepted values for the preparation of an optimum SNV formulation [36,41]. The high EE values are attributed to the lipophilicity and poor aqueous solubility of the drug. Previous studies have demonstrated that the greater the lipophilicity of the spanlastic nano-vesicles is, the higher the EE of the lipophilic drug is [29,50]. DL, on the other hand, was selected at the same level of importance as that of EE. Despite being calculated using different parameters than EE, DL is affected by the EE values of the formulation. This is suggested to take place because both responses are calculated using the amount of the entrapped drug as a key parameter. The higher the entrapped amount of the drug, the higher the values of EE and DL are [32].

Zeta potential was selected as a parameter of less importance than EE and DL despite its importance in indicating the stability of the formulation. This was because all formulations in the study showed high ζ values (Table 1). It was also reported that the use of tweens as edge activators helps increase the stearic stabilization of the nanocarriers [51,52]. Combined with the stabilizing characteristics of tweens, the high values of all SNV formulations prepared in this study indicated to a great extent that the nanoparticles were stable during storage with a minor tendency to aggregate. This stood behind the reason for which ζ was selected as a less important parameter in designing the FOSNV as compared to the PS, EE, and DL. Amongst all the studied parameters, the PDI emerged as the least important one included in the design of the FOSNV formulation. This was attributed to the findings that all formulations in this study exhibited a PDI value less than 0.5, indicating a monodisperse particle population with narrow particle size distribution.

### 3.7. FOSNV Physicochemical Characteristics

As shown by Table 2, the prepared FOSNV manifested excellent physicochemical characteristics. The FOSNV manifested a PS of less than 200 nm. This indicated a high potential for enhanced penetration and increased FMT bioavailability. It was reported that the primary mechanism by which any cell takes in small chemicals and particles is endocytosis, in addition to other mechanisms. Using ATP as an energy source, endocytosis is the process by which items are actively transported into the cell by being engulfed in its phospholipid bilayer [53]. According to reports, pinocytosis and phagocytosis are the two primary endocytosis processes. The primary physicochemical characteristics of nanocarrier systems that affect endocytosis-dependent cellular uptake are the particle size and PDI. Particles with a PS less than 200 nm can penetrate easily through GIT via transcellular uptake (endocytosis and pinocytosis) and paracellular uptake [54,55,56]. This justifies the reason that nanocarriers within the particle size range of 30–200 nm would possess a great potential for enhancing the bioavailability of the incorporated drug. Regarding EE and DL, as stated earlier, the values describing these characteristics were measured to be appropriate for practical use of the FOSNV and enhancing bioavailability as well.

The zeta potential of colloidal nanoparticles could be identified as the total surface charges of the nanocarrier. The negative charges on the surface of the FOSNV are suggested to be a result of the polar heads of S 60 (partially negative) oriented towards the external aqueous phase [57]. With regard to deformability, it was reported that deformable vesicles differ from other traditional firm vesicles in having a higher capability of crossing biological membranes due to their elastic nature, which is a crucial feature [34,58]. The FOSNV manifested a high deformability index (Table 2), which indicated high elasticity. Increasing the elastic character of these vesicles with an EA (tween 60), it would be possible for even large vesicles to pass through the cell membranes, resulting in enhanced drug permeability [44]. Moreover, using hydrophilic surfactants with high HLB values (tween 60, 14.9) can destabilize vesicular membranes and result in systems with varying degrees of deformability. These flexible vesicles could squeeze themselves through pores that are smaller than their widths under the control of the water gradient [34,59].

### 3.8. Differential Scanning Calorimetry

Comparing the thermograms of the pure drug, plain optimized SNV, and FOSNV revealed the disappearance of both the endothermic peaks representing the melting of FMT and S 60. This indicated that the drug was completely soluble in the vesicles. In addition, the disappearance of the endothermic peak of S 60 indicated that the surface-active agent was not present in a crystalline or solid state. This was reported in other publications [39,60]. In addition, no new peaks were observed. This indicated that the drug and all the excipients used in formulating the FOSNV were compatible, i.e., no physical interaction between the drug and excipients was detected [39].

### 3.9. Morphological Characteristics of FOSNV

The TEM revealed that the FOSNV were unilamellar nano-vesicles (Figure 7). The optimum spanlastic nano-vesicle population exhibited no aggregation of particles. This could be explained on the basis of the effect of the dense negative charges on the FOSNV surfaces (as evidenced by appropriate ζ value) and the stearic effect of tween 60 [51,52,57].

Moreover, the PS of the FOSNV determined using the TEM, 117.5 ± 17.08 nm, was significantly smaller than the size determined by dynamic light scattering technique (170.6 ± 4.57 nm). Typically, a smaller size is detected from the TEM, and this is commonly explained by the samples’ dry state. Due to the particles’ hydrated state, DLS measurements, on the contrary, reveal a larger size as revealed in past studies who found the same results [41,61].

### 3.10. In Vitro Drug Release Study

The fast release of FMT from FMT suspension was observed in the release study. This was attributed to, at least partially, the use of a surface-active agent to maintain sink conditions. Achieving sink conditions through using a high volume of dissolution medium suffers the drawback of very low released drug concentration, which makes the test inadequate for testing drug release in early time points, especially for controlled release drug delivery systems (where the early released drug concentrations are too low) [62]. The drug release from a suspension requires the dissolution of the drug from the solid particle to form a saturated solution followed by the diffusion of the drug from the saturated solution inside the cellulose bag into the bulk dissolution medium [63,64]. The use of surfactant in the bulk dissolution medium results in enhanced diffusion of the drug, which yields a pattern of very fast drug release from the pure drug suspension as noticed in this study. This could be limited to the dissolution-dependent drug release [63]. The release of FMT from the FOSNV was shown to be of different pattern that suggests a different mechanism of release. FMT release from the FOSNV was shown to be biphasic with a very fast release in the beginning followed by a controlled release. The fast release could be explained by the sight of the molecules of the drug that remain adsorbed on/near the surface of the nano-vesicles. These superficial drug molecules are ready for dissolution and detach from the vesicles or escape the vesicular membrane very fast [32,65]. The control of FMT release was achieved as a result of many factors, including (a) the elevated affinity of the drug molecule to the vesicular membrane components as compared to affinity to the external phase, (b) the poor solubility of FMT in the external phase, and the diffusion of the drug through the FOSNV membrane [63,66,67]. This makes the process of controlled FMT release from the FOSNV a diffusion-dependent process.

### 3.11. In Vivo Pharmacokinetic Study

Despite being superior to FMT suspension or to the simple solubilization of FMT by any solvent (due to the biphasic, controlled release pattern), the SNV could help achieve advanced drug delivery for BCS class II drugs with minor advantage to BCS class IV ones. This is because the advanced release of the latter ones would not help improve the permeability of the drugs. Spanlastic nano-vesicles were reported to enhance the permeability of drugs through GIT as well as skin and other biological barriers in addition to enhancing the dissolution of the drugs [19,21,25]. This was the reason lying behind the selection of this drug delivery system to formulate FMT, which is a model for BCS class IV. The mechanism by which the FOSNV manifested high permeability is suggested to be correlated to the flexibility of the nano-vesicles. This was reported by many researchers [15,18,50,68,69]. In a study published by Abdelbari et al., a strong correlation between the permeability of the spanlastic nano-vesicles and flexibility was reported [50]. Another study conducted by Magdy et al. also reported that as the deformability of the vesicles increased, the permeability of the nano-vesicles increased consequently [69]. The enhanced FOSNV permeability is suggested to be attributed to the flexibility and high deformability of the vesicles. They are suggested to have the ability to squeeze themselves and enable transport through the narrow pores and hence deliver the cargo efficiently [15,50,68]. The vesicular flexibility and ultradeformability is attributed, at least in part, to the edge activator, which increases membrane fluidity, resulting in the SNVs being highly curved without being ruptured [50,69,70].

The pharmacokinetic behavior of FMT following the administration of a sublingual liquid of the FOSNV in comparison to the FMT suspension was detected using certain pharmacokinetic parameters. These parameters included C_max_, T_max_, AUC_0–24_, and F_re_ (Table 3). Comparing the FOSNV to the FMT suspension as a reference revealed that the C_max_ of the FOSNV was significantly higher than that of the FMT suspension by 19%. Since C_max_ represents the highest concentration of the drug in plasma, it reflects the intensity of the effect of the drug action. This indicates that a spanlastic nano-vesicle could help improve C_max_ and enhance the efficacy of FMT. On the other hand, T_max_ was shown to be less in FMT suspension compared to the FOSNV. This indicated that the drug achieved its highest concentration in a faster time point when administered as a suspension rather than the FOSNV. This could be explained by referring to the pattern of drug release (Figure 8). It was shown that the FOSNV released FMT in a fast onset followed by controlled release; however, the FMT suspension caused the drug to be released at a faster rate, so it was not surprising that FMT reached its maximum concentration in blood rapidly following the FMT suspension administration compared to FOSNV administration. This also indicated that the in vivo pharmacokinetic behavior correlated well with the in vitro drug release study. However, the difference in T_max_ value regarding the two formulations under investigation, despite being statistically significant, was not of practical importance. This could be illustrated by stating that the minimum effective concentration (MEC) of FMT was reported to be 0.2 μg/mL [71]. Both formulations exceeded the MEC of FMT in blood during the first 15 min, increasing the significance of the practical importance of T_max_ difference [32].

The AUC indicates the availability of the drug in the blood. When used in orally administered drugs, it reflects the extent of the absorption of the drug following the administration of certain formulations [72]. As it was shown by Table 3, the administration of FMT in the FOSNV caused a 14% increase in the AUC_0–24_ compared to the FMT suspension. This could be great evidence on the increased membrane permeability of the FOSNV. Absolute bioavailability (calculated by comparing the AUC of the two formulations administered via the same route) was also shown to be 114.086%. This indicated that the FOSNV increased the bioavailability of the drug due to enhanced membrane permeability. The increased deformability and elasticity of SNVs induced by the presence of EAs, which boosted the fluidity of the vesicular membrane and allow for the passage of SNVs through tiny pores in biological membranes, may also be used to explain the higher bioavailability of the FOSNV [73]. As a result, spanlastic nano-vesicles enhanced FMT membrane permeability due to high flexibility and deformability.

### 3.12. Constraints and Future Research

Potential limitations for this study can be identified to correlate to safety issues of the administration of spanlastics. The safety of spanlastic administration via different routes of administration was not addressed properly, neither in any previous research work nor in this study. Future research is required to assess safety, toxicity, and possible adverse reactions upon the administration of spanlastic nano-vesicles. In addition, the incorporation of the FOSNV into a muco-adhesive drug delivery system to increase their sublingual retention time, and the assessment of the effects of this incorporation on the characteristics of the FOSNV is required to be conducted in an extension for this work.

## 4. Materials and Methods

### 4.1. Materials

EIPICO Pharmaceutical Co. (10th of Ramadan, Sharkia, Egypt) generously gifted FMT. Tween 60, tween 80, span 60, and disodium hydrogen phosphate were supplied by Oxford Lab Fine Chemicals, LLP. (Maharashtra, India), and polyoxyl 40 hydrogenated castor oil was bought from LANXESS energizing Chemistry (Cologne, Germany). Dialysis membrane with molecular weight cutoff value of 12,000–14,000 Dalton was supplied by FREY Scientific, NH, USA. Sodium lauryl sulfate (SLS) and potassium dihydrogen phosphate were purchased from EL-NASR pharmaceuticals (Cairo, Egypt).

### 4.2. Methods

#### 4.2.1. Preparation of SNVs

SNVs were prepared using ethanol injection method [39]. Span 60 and tween 60 were utilized as the non-ionic surface-active agent and the EA, respectively. Span 60 was dissolved completely in enough ethanol to make 10 mL of the ethanolic solution. The ethanolic solution was injected into a preheated aqueous solution (60 °C) of T 60 at a speed of 0.2 mL/min (with a phase volume ratio of 1:1). The ethanolic injection into the aqueous solution took place while the aqueous phase was kept under stirring at 800 rpm using a magnetic stirrer (MSH-20A, Witeg Labortechnik GmbH, Wertheim, Germany). The preparation was stirred for 30 min after the injection of the ethanolic solution was performed. After cooling down to room temperature, the preparation was sonicated (Fisherbrand™ 11201, Thermo Fisher Scientific Inc., Waltham, MA, USA) for 10 min. Finally, the spanlastic formulations were then kept at 4 °C for an overnight period to allow SNVs to fully mature.

#### 4.2.2. Experimental Design

Preliminary studies were conducted to detect the most suitable ingredients with the best concentration ranges for formulating SNVs. In these studies, span 40, span 60, and span 80 were used as the non-ionic surface-active agents. Tween 20, tween 40, tween 60, and tween 80 were utilized as the edge activators. Span 40 failed to produce vesicles, while span 80 resulted in vesicles with large particle sizes. It was clearly detected that span 60 and tween 60 made the best combination to formulate SNVs with appropriate characteristics. The concentration ranges of the used ingredients were also tested in the preliminary studies. The best concentration ranges to formulate the most adequate SNVs were selected based on the preliminary studies and were utilized in the experimental design for optimizing the SNV formulation. Box–Behnken design (BBD) was used to optimize spanlastics (Stat-Ease, MN, USA, Design Expert^®^ software, version 11.1.2.0). The Box–Behnken design has the benefit of highlighting the difficulties with the experimental constraints and avoiding superfluous combinations of treatments [74]. The independent variables that showed the highest impact on the physicochemical characteristics of SNVs in the preliminary studies were the concentrations of S 60 and T 80 and the amount of FMT initially incorporated into the SNVs. Span 60 was used at concentration levels of 10, 15, and 20 mg/mL, while the concentrations of tween 60 used were 5, 7.5, and 10 mg/mL. The amounts of FMT incorporated into the SNVs were 1, 1.5, and 2 mg. A summary of the experimental design is listed in Table 4. Particle size (PS), polydispersity index (PDI), zeta potential (ζ), entrapment efficiency (EE), and drug loading (DL) were used as the dependent variables that would be affected by the change in independent variable levels. These dependent variables affect extensively the functionality of the SNVs’ ability to deliver FMT efficiently [75]. This design gave rise to 15 formulae. The actual compositions of these formulations are listed in Table 5.

The surface response method was utilized to obtain the optimum formulation with the highly desired physicochemical characteristics. Analysis of values of dependent variables has led to determining the effect of the concentrations of S 60 and T 80 and the amount of FMT initially added on the physicochemical characteristics of the prepared SNVs. An optimum SNV formula could then be deduced from the statistical analysis of the detected values of the dependent variables in relation to the changes in the independent variables’ values. This was calculated using Design Expert^®^ software, and a number of optimum formulae were suggested. The SNV formula with desired physicochemical characteristics was proposed to have the minimum PS and PDI and maximum ζ, EE, and DL. In order to evaluate the optimized formula, the actual values of the dependent variables were also compared with their expected values by calculating the percentage relative error in the principle outlined below [76]:

(5) $$\;\%\;relative\;error=\frac{predicted\;value-observed\;value}{predicted\;value}\times 100$$

#### 4.2.3. Determination of Particle Size, Polydispersity Index, and Zeta Potential

Particle size, polydispersity index, and zeta potential were measured using Zetasizer^®^ (Instrument Malvern Ltd., Worcestershire, UK). Samples were adequately diluted with deionized water to obtain the appropriate magnitude of scattering at 25 °C. After being properly placed in the cuvette, the mean PS of the sample population was detected using the dynamic light scattering (DLS) technique. PDI was calculated from the determined PS of the whole particle population. By detecting the mobility of charged vesicles during electrophoresis in an electrical field while using deionized water, ζ was determined [34]. All measurements were conducted in triplicates, and data were represented as mean ± standard deviation.

#### 4.2.4. Determination of Entrapment Efficiency and Drug Loading

EE and DL of FMT in the spanlastic nano-vesicles were determined by the indirect method. The indirect method comprises the determination of the entrapped drug inside the SNVs through measuring the amount of the free drug in the external aqueous phase [32]. Aliquots of samples, each 2 mL in volume, were centrifuged by the aid of a cooling centrifuge (Hermle Labortechnik GmbH, Wehingen, Germany) at 17,000 rpm and 4 °C for 2 h [19,32]. After centrifugation, separation of the SNVs dispersion into sediment (containing SNVs) and clear supernatant took place. The supernatant was decanted and filtered using a 0.22 µ filter. The free amount of FMT in the supernatant was detected using the spectrophotometric method at 282 nm after suitable dilution. EE was determined using the following equation [32,46,77]:

(6) $$EE(\%)=\frac{Total\;amount\;of\;FMT-Free\;amount\;of\;FMT}{Total\;amount\;of\;FMT}\times 100$$

The drug-loading percentage was also calculated using the free and totally incorporated amounts of FMT. However, DL compares the amount of the FMT entrapped inside the SNVs to the total amount of the SNVs. DL was calculated using the following equation [32,46]:

(7) $$\;DL(\%)=\frac{Total\;amount\;of\;FMT-Free\;amount\;of\;FMT}{Total\;amount\;of\;all\;SNVs\;components}\times \;100$$

#### 4.2.5. Measurement of Spanlastic Nano-Vesicle Deformability

The deformability index (DI) is used as a measure of the elasticity of the SNVs. The extrusion process was used to characterize DI of the optimum SNV [68]. The optimum spanlastic nano-vesicle was extruded through a membrane filter (nylon membrane, 100-nm pore size) under constant vacuum pressure for ten minutes. The weight of the sample extruded in the specified time and the mean PS of the SNV after extrusion were determined. DI could be calculated using the equation below [68]:

(8) $$DI={J\left(\frac{r_{v}}{r_{p}}\right)}^{2}$$

where J is the weight of sample (g) extruded by membrane filter in 10 min, r_v_ stands for the size of the spanlastic nano-vesicles after extrusion, and r_p_ represents the pore size of the membrane filter used.

#### 4.2.6. Differential Scanning Calorimetry (DSC)

Various samples (1–3 mg each) of FMT, the lyophilized optimized spanlastic nano-vesicle formula, and the corresponding plain (drug-free) spanlastic formula were heated in a sealed aluminum 40 µL pan from 0 to 300 °C with a scanning rate of 10 °C/min (Shimadzu DSC 60, Kyoto, Japan). Changes in the energy consumption as a result of changing temperatures were recorded, and thermograms were constructed [19].

#### 4.2.7. Morphological Characterization

The morphology of the optimized FMT-loaded spanlastics was observed using a transmission electron microscope (JEM-1400, Jeol, Tokyo, Japan) running at 80 kV. The carbon-coated copper grid was topped with one drop of the vesicular suspension. Prior to inspection, the grid was allowed to dry at room temperature. This measurement was performed at the National Institute for Research, Cairo, Egypt [41,77].

#### 4.2.8. In Vitro FMT Release Study

The in vitro drug release study of FMT from FMT-loaded optimum SNV in comparison to FMT suspension was conducted utilizing the diffusion through dialysis bag method. The study was conducted using a USP dissolution tester, apparatus type II (DIS 800i, Copley, Nottingham, UK). The dialysis membrane was cut into suitable pieces and immersed in phosphate buffer solution (pH 6.8) 24 h prior to the start of the test. Each piece was sealed from one end, leaving the other end open for loading of the optimum SNV. After placing the optimum SNV formulation (equivalent to 5 mg FMT) inside the dialysis bag, the other end was sealed, and the whole bag was checked for any leakage. The release medium was composed of a preheated (37 °C ± 0.5 °C) 250 mL PBS, pH 6.8, to simulate salivary fluid. Dissolution medium contained sodium lauryl sulfate at a concentration of 0.5% *w*/*v* to aid the dissolution of the drug and create sink conditions [75]. The speed of rotation was set at 50 rpm. At predetermined time points (ranging from 15 min to 24 h), 5 mL samples were withdrawn and replenished with preheated dissolution medium of the same volume. The concentration of FMT was determined spectrophotometrically at 285 nm, as previously stated.

#### 4.2.9. Pharmacokinetic Study

##### Animals

The in vivo study was performed on male Wistar rats. Rats were purchased from the National Research Center (Dokki, Giza, Egypt). Rats selected for study ranged in weight from 190 g to 200 g. The protocol of the in vivo study was approved by the research ethics committee at the pharmacy school, Suez Canal University, Ismailia, Egypt, and was denoted the code 202010M3.

Animals were housed in separate cages located in the animal house at the Faculty of Pharmacy, Suez Canal University. Before conducting the experiment, the rats were placed under typical living conditions for 14 days in the animal house before the experiment for acclimatization. They had free access to food (a standard lab meal that was adequate in terms of nutrients) and water with 12 h day/night cycle maintenance. The animal rooms were maintained at 50% relative humidity and kept at a temperature of 25 ± 2 °C [32].

##### Test Protocol

Animals were divided into two groups, each containing 6 animals. The first group contained the animals that will be given FMT SNV, while FMT suspension was administered to the second group of rats. FMT was given to the rats in a dose of 5 mg/kg/day through the sublingual route. This route was chosen particularly to avoid any possible degradation of the drug by gastric fluids [25]. To compensate for the impact of various treatments on the weight of the rats in each group, the weight of the rats was noted both before and after different treatments.

Following administering FMT formulations, blood samples were taken from the tail vein into tubes containing heparin at (0, 0.25, 0.5, 1, 2, 4, 8, 12, and 24 h). Samples were centrifuged for 10 min at 15,000 rpm, and 100 µL plasma samples were withdrawn. Samples were analyzed for the concentration of FMT using HPLC.

##### HPLC Quantification of FMT

HPLC techniques comprise the analysis and separation techniques. It is generally used whenever analysis of a mixed solution is required. The detection of FMT concentration using the HPLC method followed the following procedure. In a 10 mL volumetric flask, an aqueous solution of famotidine was added with a final concentration range of 2.5–12.0 µg/mL. An amount of 20 μL of the solution was injected into a Phenomenex^®^ C18 column (5 µm pore size with 150 4.6 mm id; Phenomenex, Torrance, CA, USA) and was isocratically eluted using a mobile phase of methanol–water–acetonitrile and THF (40: 40: 16: 4 *v*/*v*) at a flow rate of 1 mL/min. The drug concentration was detected using UV detector at 270 nm.

##### Detection of Pharmacokinetic Behavior

The plasma drug concentration–time curve for the FOSNV and FMT suspension (used as a reference) was constructed through plotting the time as the independent variable (on *X*-axis) and plasma drug concentration as the dependent variable (on *Y*-axis). Five pharmacokinetic parameters were used to detect the pharmacokinetic parameters of the FOSNV formulation and the reference drug suspension. These parameters included maximum drug concentration (C_max_), time for maximum drug concentration (T_max_), mean retention time (MRT), area under the curve for the test period (AUC_0–24_), and relative bioavailability (F) of the test formulation as compared to the reference pure suspension [78]. Data were analyzed using compartmental analysis of the PKsolver^®^ 2.0 analysis tool.

##### Statistical Analysis

All data were presented as mean (n = 3) ± standard deviation. The statistical evaluation of the data was performed using one-way ANOVA testing followed by a post hoc test at a *p*-value less than 0.05 with the aid of SPSS^®^ 23.0 software (IBM, Chicago, IL, USA). Analyzing the data of the two groups used in the in vivo pharmacokinetics study was performed using the Student’s *t*-test.

## 5. Conclusions

Regarding FMT as a model drug for BCS class IV, FMT was investigated to face many hurdles that retard and delay or even reduce its bioavailability upon oral administration. FMT, being a BCS class IV member, exhibited poor aqueous solubility and poor penetration. In addition, the molecule manifested accelerated degradation in acid medium. Moreover, the pharmacological indication and short half-life time necessitated that the pattern of the drug release could be optimized if it allows for a combination of a fast release and a controlled release of the drug. These characteristics combined made the delivery of FMT a challenging process. Spanlastic nano-vesicles manifested excellent characteristics that enable them to be utilized for specific tailor-made drug delivery. SNVs as nano-based drug deliver carriers demonstrated high ability to enhance drug dissolution, high flexibility that improved membrane permeation, and promising release characteristics enabling a combination of fast release to establish a rapid onset of action followed by controlled release to compensate for the elevated drug clearance and low half-life time. A single problem yet needed to be solved is the gastric instability caused by acid-accelerated degradation. This matter could be fixed via the administration of the FOSNV as a sublingual fluid. SNVs showed great versatility to be used via peroral, buccal, sublingual, or transdermal routes of administration. This allowed the FOSNV to avoid the peroral route of administration, hence bypassing the potential of the acid-catalyzed degradation of FMT. In summary, spanlastic nano-vesicles were represented as a solution to overcome many obstacles facing the delivery of BCS class II and class IV drugs with a tailor-made enhanced release and pharmacokinetic behavior. However, safety, effects on different tissues, and the scale-up studies regarding the spanlastic nano-vesicles are not fully investigated. Being surfactant-based nano-vesicles, spanlastics may have an impact on soft tissues, which might restrict their use via some routes of administration, like I.V., I.M., or S.C. injection. This also requires more investigations. As an extension for this study, the incorporation of the FOSNV into a sublingual mucoadhesive drug delivery system will be investigated to curtail the impact of saliva and tongue movement on the retention of this drug delivery system in the region of application.

## Figures and Tables

**Table 1:** The layout of the experimental design responses revealing dependent variables of SNVs.

F Code	PS (nm)	PDI	ζ (mV)	EE (%)	DL (%)
F1	185.17 ± 4.01	0.491 ± 0.01	−33.77 ± 0.12	71.62 ± 0.66	3.46 ± 0.03
F2	247.50 ± 0.66	0.435 ± 0.01	−36.17 ± 0.84	76.14 ± 0.32	5.74 ± 0.02
F3	217.97 ± 9.06	0.454 ± 0.05	−35.93 ± 3.33	75.85 ± 0.86	7.98 ± 0.08
F4	259.13 ± 9.48	0.409 ± 0.00	−39.50 ± 1.31	77.43 ± 0.60	7.19 ± 0.05
F5	89.04 ± 0.57	0.384 ± 0.02	−24.83 ± 3.99	59.37 ± 1.84	3.28 ± 0.10
F6	163.33 ± 4.61	0.447 ± 0.01	−34.53 ± 2.61	70.64 ± 0.55	4.50 ± 0.03
F7	153.43 ± 1.40	0.378 ± 0.03	−28.53 ± 0.40	68.69 ± 0.87	6.43 ± 0.08
F8	112.37 ± 1.30	0.375 ± 0.04	−28.07 ± 0.80	65.03 ± 1.58	2.31 ± 0.05
F9	164.63 ± 4.57	0.442 ± 0.02	−33.33 ± 1.87	68.02 ± 0.80	4.34 ± 0.05
F10	133.60 ± 5.82	0.359 ± 0.04	−28.3 ± 1.51	66.33 ± 1.03	4.74 ± 0.07
F11	163.63 ± 0.35	0.428 ± 0.02	−29.13 ± 1.05	69.00 ± 0.68	4.40 ± 0.04
F12	282.20 ± 6.22	0.493 ± 0.03	−39.77 ± 0.77	82.32 ± 1.74	5.65 ± 0.11
F13	190.47 ± 4.36	0.346 ± 0.02	−35.20 ± 1.10	73.14 ± 0.69	4.20 ± 0.04
F14	100.00 ± 0.20	0.373 ± 0.01	−26.00 ± 2.43	61.52 ± 1.20	2.40 ± 0.05
F15	106.63 ± 2.40	0.389 ± 001	−27.53 ± 1.63	63.30 ± 1.65	3.07 ± 0.08

All measurements were performed in triplicates, and data are presented as mean ± SD.

**Table 2:** Physicochemical properties of the FOSNV (observed vs. predicted) with the relative errors in these values.

Characteristic	Predicted	Observed	Relative Error (%)
PS (nm)	156.71	170.58 ± 4.48	8.85
PDI	0.405	0.368 ± 0.04	10.05
ζ (mV)	−30.93	−30.82 ± 1.95	0.36
EE (%)	68.91	68.89 ± 0.48	0.03
DL (%)	6.13	6.07 ± 0.04	0.98
Deformability	-	8.26 ± 0.18	-

**Table 3:** Pharmacokinetic parameters of FOSNV formulation and FMT pure suspension.

Pharmacokinetic Parameter	FMT Suspension	FOSNV
C_max_ (µg/mL) *	2.394 ± 0.025	2.845 ± 0.151
T_max_ (h) *	2.000 ± 0.000	4.000 ± 0.000
AUC_0–24_ (μg/mL.h) *	36.447 ± 0.821	41.581 ± 0.765
F_re_ (%) *	-	114.086

* Significantly different values at *p* < 0.05.

**Table 4:** Box–Behnken experimental design using Design expert^®^ software.

Independent Variables (Factors)	Levels			Units
	Low (−1)	Medium (0)	High (+1)	
X_1_: Span 60 concentration	10	15	20	mg/mL
X_2_: Tween 60 concentration	5	7.5	10	mg/mL
X_3_: FMT amount	10	15	20	mg
**Dependent variables (Responses)**	**Units**		**Desirability Constraints**	
Y_1_: Particle size (PS)	nm		Minimize	
Y_2_: Polydispersity index (PDI)			Minimize	
Y_3_: Zeta potential (ζ)	mV		Maximize	
Y_2_: Entrapment efficiency (EE)	%		Maximize	
Y_4_: Drug loading (DL)	%		Maximize	

**Table 5:** Compositions of SNV formulations as proposed by experimental design software.

Formulation Code	Independent Variables		
	Span 60 Concentration (mg/mL)	Tween 60 Concentration (mg/mL)	FMT Amount (mg)
F1	20	10	15
F2	15	10	20
F3	10	7.5	20
F4	15	5	20
F5	10	7.5	10
F6	15	7.5	15
F7	10	5	15
F8	20	7.5	10
F9	15	7.5	15
F10	10	10	15
F11	15	7.5	15
F12	20	7.5	20
F13	20	5	15
F14	15	10	10
F15	15	5	10

## Data Availability

The original contributions presented in this study are included in the article. Further inquiries can be directed to the corresponding authors.
